# Thermal behavior of a two-story concrete building under controlled winter and heat wave scenarios in the sense-city equipment through temperature, flux and energy consumption dataset

**DOI:** 10.1016/j.dib.2020.106458

**Published:** 2020-10-23

**Authors:** Zohra Djatouti, Julien Waeytens, Ludovic Chamoin, Patrice Chatellier

**Affiliations:** a Univ. Gustave Eiffel, 14-20 Bd Newton, F-77447 Marne-la-Vallée cedex 2, France; b Université Paris-Saclay, ENS Paris-Saclay, CNRS, LMT, 4 des Sciences, F-91190 Gif-sur-Yvette, France

**Keywords:** Thermal building, Controlled climatic scenarios, Sensor outputs, Sense-city, Inverse analysis, Data assimilation

## Abstract

The article describes thermal datasets collected in a two-story concrete building of the Sense-City equipment during various controlled climatic scenarios. Using the Sense-City climatic chamber, we reproduced stationary thermal conditions, a typical winter climate of the south of France and Paris 2003 heat wave. Each of the three scenarios has a duration of about one week. The datasets contain temperature, heat flux and energy consumption sensor outputs. In [1], the stationary conditions data were exploited for an experimental identification of thermal characteristics of the building whereas the winter and the heat wave data were used in a goal-oriented model updating technique. The datasets can also be useful to validate modeling and simulation.

## Specifications Table

**Table:** 

Subject	Engineering (General)
Specific subject area	Thermal building behavior
Type of data	Table Figure Plan
How data were acquired	The used sensors are as the following: • Temperature in the rooms and in the soil: PT100 4-wires probes (model: PT100 class B from Minco and TC S.A.) • Temperature on surfaces of the building (walls, floors): PT100 4-wires surface probes (model: PT100 class B from Minco and TC S.A.) • Outdoor temperature: weather station (model: weather transmitter WXT536 Vaisala) • Heat flux through wall surfaces: standard heat flux sensor (model: CAPTEC, dimensions: 300mm×300mm, thickness of about 0.5mm, characteristics: sensitivity higher than $150\;\mu V/(W/m^{2})$, copper surface) • Energy power consumption of heaters connected on electrical outlets: Wi-Fi smart switch remote control socket (model: Basic R2 sonoff)
Data format	Raw
Parameters for data collection	The sensor outputs were collected from a two-story building under controlled climatic scenarios using the Sense-City climatic chamber. No human occupancy in the building and no sun light were considered in the experiments. The measurement was performed with a time step of 15 min.
Description of data collection	All the sensors were plugged into a PEGASE data acquisition card that sent the measured data to a web server through a WiFi connection.
Data source location	Institution: Université Gustave Eiffel City/Town/Region: Champs sur Marne Country: FRANCE Latitude and longitude (and GPS coordinates, if possible) for collected samples/data: 48.84178, 2.58972
Data accessibility	With the article
Related research article	Z. Djatouti, J. Waeytens, L. Chamoin, P. Chatellier, Goal-oriented sensor placement and model updating strategies applied to a real building in the Sense-City equipment under controlled winter and heat wave scenarios, Energy & Buildings, DOI:10.1016/j.enbuild.2020.110486.

## Value of the Data

• The thermal behavior of the two-story building was monitored using more than 30 sensors for three different climates (stationary conditions, winter and heat wave) under fully controlled conditions in a climatic chamber.
• These data can be valuable for researchers, engineers and students.
• The thermal datasets can be used to test methods for the identification of thermal building characteristics such as the thermal resistance of walls. It can also be interesting for the validation and the calibration of building thermal models and simulation software.
• The datasets can be exploited in practical sessions for master student classes. For example, students can build the building numerical mock-up, select a thermal building model and compare the numerical results with the experimental data.
• These data can help us improve our understanding of the thermal behavior of buildings. This understanding is very useful in the context of global warming.

## 1 Data Description

**Fig. 1:**
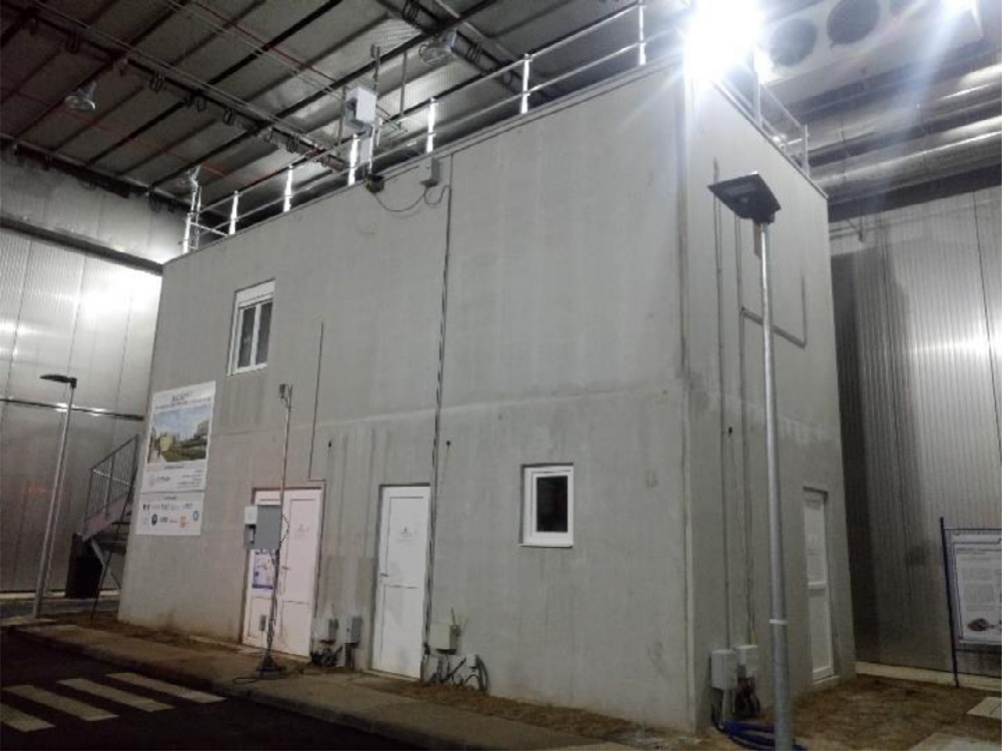
Picture of the two-story building in the Sense-City climatic chamber.

Fig. 1 shows a picture of the two-story building of the Sense-City equipment, which is located on the campus of Université Gustave Eiffel at Champs-sur-Marne (France). It has a $40\;m^{2}$area (10m×4m) and 6mheight.

**Fig. 2:**
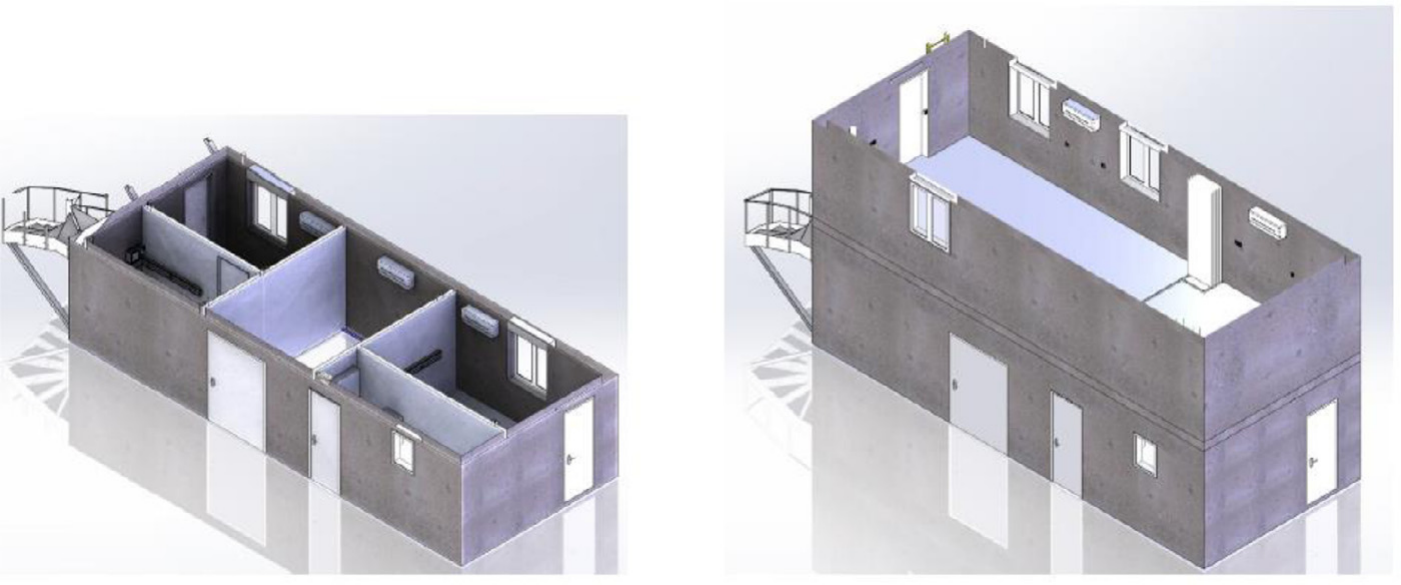
A 3D view of the ground (left) and the first floor (right) of the studied building, Figure modified from the original research article [1].

Fig. 2 represents a 3D numerical mock-up of the building. The ground floor, composed of 5 rooms, is given on the left in Fig. 2. The first floor, which corresponds to an open-space with two offices (named “Office 1″ and “Office 2), is illustrated on the right in Fig. 2. In the experiments, the removable wall was not installed to separate the two offices in the first floor.

The access to the first floor is provided by an outside staircase.

The detailed map of the building is given in the supplementary file “Building_plan.pdf”. The building is made of reinforced concrete without insulation. Its envelope (walls and floors) is only made of 200mmprecast concrete walls. The first floor consists of 5 rooms (see top of Fig. 2 and the building map in the supplementary “Building_plan.pdf”). They are all of different areas and usages (see Table 1) and are separated by 72mmthick concrete walls. On the first floor, the so-called “GF1” and “GF3” rooms are equipped with a geothermal heating floor that can be fueled by a heat pump. The second floor is an open-space with two offices (Office 1 and Office 2). The building is equipped with two controlled mechanical ventilation (CMV) systems (simple and double flow) and an air conditioning system. For our application, only the simple-flow CMV was used.

**Table 1:** Use and area of the two-story building rooms.

Room	Area ($m^{2})$	Use
GF1	9.2	Optical fiber
GF2	9.9	Water loop
GF3	10.5	Heat pump
GF4	4	Technical room
Office	34.6	Open-space

**Fig. 3:**
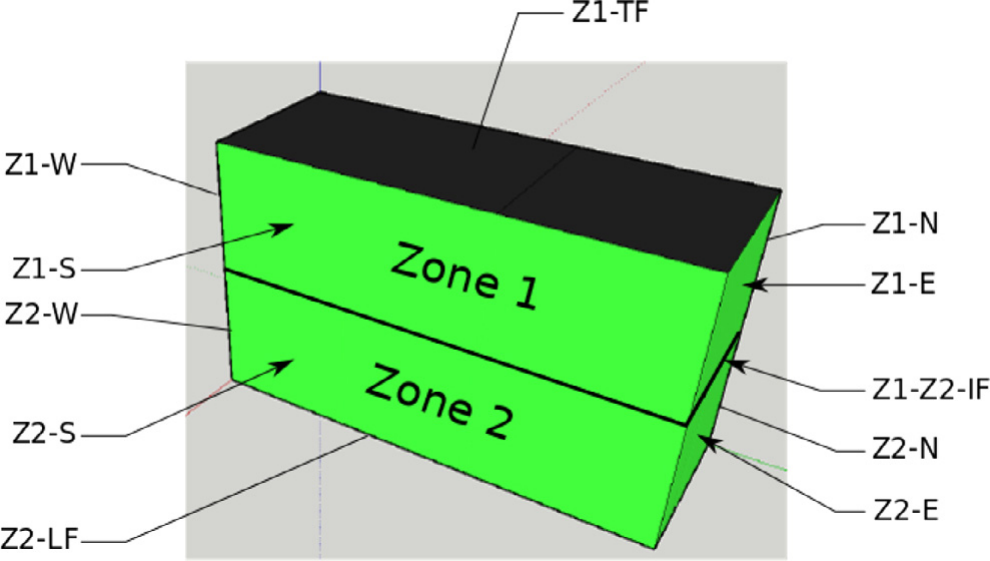
Notations of zones, walls and floors of the building, Figure modified from the original research article [1].

In [1], we considered a two-zone physical model to describe the thermal behavior of the building. The zones, walls and floors notations, e.g. “Z1-N” (North oriented wall in Zone 1) and “Z1-Z2-IF” (Intermediate floor between Zone 1 and Zone 2) are defined in Fig. 3. These notations are used to reference the position of the sensors in the datasets.

**Fig. 4:**
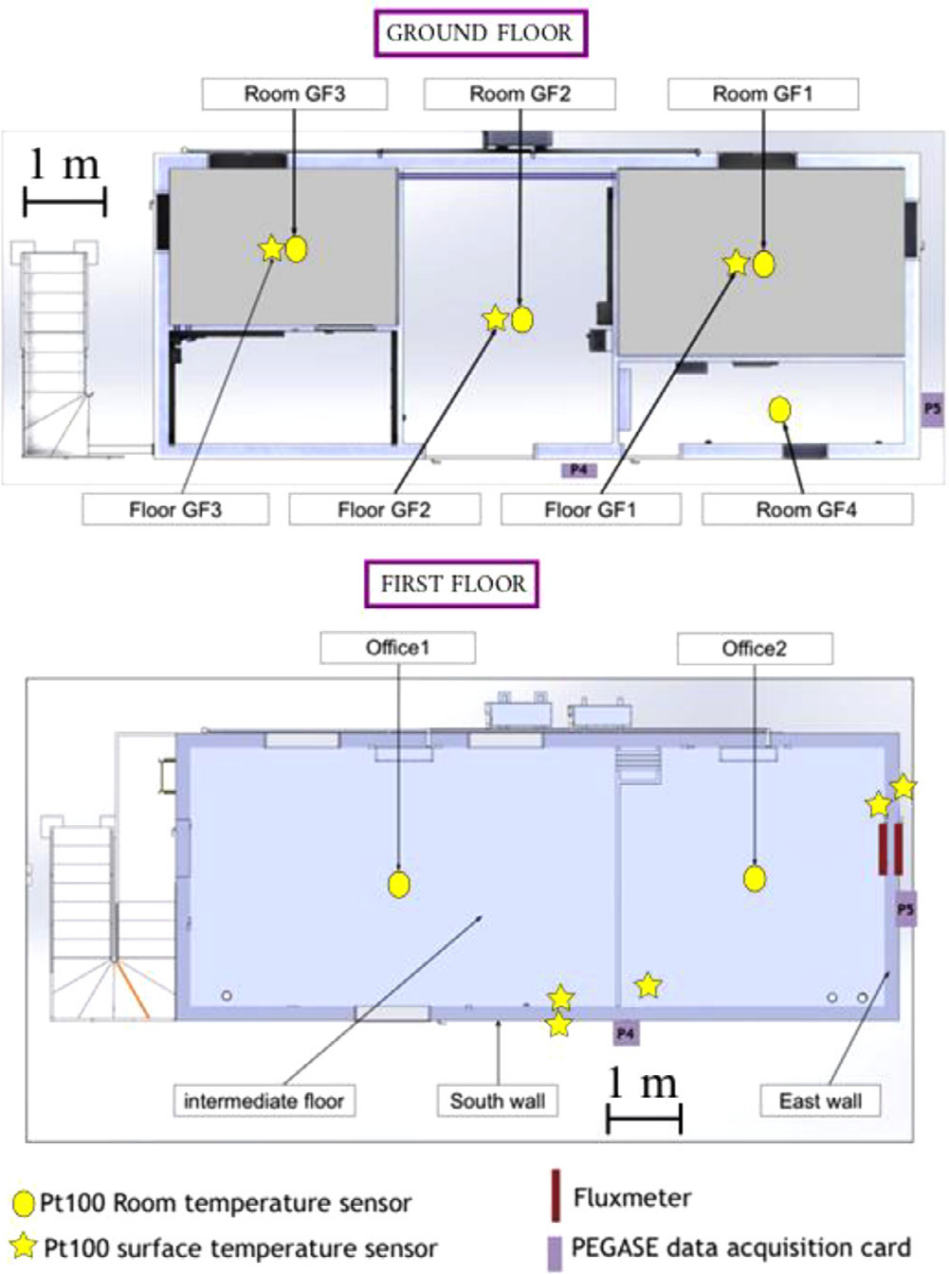
Distribution of the temperature and the heat flux sensors in the two floors of the building, Figure modified from the original research article [1].

Fig. 4 shows the distribution of the temperature and the heat flux sensors in the building. The yellow circles correspond to sensors for the monitoring of the ambient temperature of the rooms whereas yellow stars are associated with sensors to measure temperature on wall and floor surfaces. On the ground floor, 4 temperature sensors (resp. 3 temperature sensors) were used to measure the ambient temperature of the rooms “GF1”, “GF2”, “GF3” and “GF4” (resp. to measure the surface temperature on the lower floor in rooms “GF1”, “GF2” and “GF3”). On the first floor, the ambient temperature of the open-space is monitored by two temperature sensors distributed in Office 1 and in Office 2, the temperature of the inside and the outside faces of South and East oriented walls are recorded, another temperature sensor is positioned on the surface of the floor (close to the South wall) and two heat flux sensors are placed on the inside and outside faces of the East oriented wall.

Raw data associated with the stationary conditions scenario, named “Scenario 1″, are given in txt and Excel formats in the supplementary files “Data_scenario1.txt” and “Data_scenario1.xlsx”. The files contain 10 rows:

• Date: Date in Day/Month/Year Hour:Minute
• Tout ( °C): Outside temperature recorded by the weather station mounted on the top of the building
• T_office1-Z1 ( °C): Temperature in Office 1 of Zone 1
• T_office2-Z1 ( °C): Temperature in Office 2 of Zone 1
• Tsi-Z1-S ( °C): Surface Temperature on Inside face of South wall in Zone 1 (Z1-S)
• Tse-Z1-S ( °C): Surface temperature on Outside face of South wall in Zone 1 (Z1-S)
• Tsi-Z1-E ( °C): Surface Temperature on Inside face of East wall in Zone 1 (Z1-E)
• Tse-Z1-E ( °C): Surface Temperature on Outside face of East wall in Zone 1 (Z1-E)
• Flux_int_Z1-E (W/m2): Flux on Inside face of East wall in Zone 1 (Z1-E)
• Flux_ext_Z1-E (W/m2): Flux on Outside face of East wall in Zone 1 (Z1-E)

Raw data associated with the winter climate scenario, named “Scenario 2″, are given in txt and Excel formats in the supplementary files “Data_scenario2.txt” and “Data_scenario2.xlsx”. The files contain 23 rows:

• Date: Date in Day/Month/Year Hour:Minute
• Tout ( °C): Outside temperature recorded by the weather station mounted on the top of the building
• T_office1-Z1 ( °C): Temperature in Office 1 of Zone 1
• T_office2-Z1 ( °C): Temperature in Office 2 of Zone 1
• Tsi-Z1-S ( °C): Surface Temperature on Inside face of South wall in Zone 1 (Z1-S)
• Tse-Z1-S ( °C): Surface temperature on Outside face of South wall in Zone 1 (Z1-S)
• Tsi-Z1-E ( °C): Surface Temperature on Inside face of East wall in Zone 1 (Z1-E)
• Tse-Z1-E ( °C): Surface Temperature on Outside face of East wall in Zone 1 (Z1-E)
• TGF1 ( °C): Temperature in room GF1 of Zone 2
• TGF2 ( °C): Temperature in room GF2 of Zone 2
• TGF3 ( °C): Temperature in room GF3 of Zone 2
• TGF4 ( °C): Temperature in room GF4 of Zone 2
• Tslf_GF1 ( °C): Surface Temperature on the face of the Lower Floor (LF) in room GF1
• Tslf_GF2 ( °C): Surface Temperature on the face of the Lower Floor (LF) in room GF2
• Tslf_GF3 ( °C): Surface Temperature on the face of the Lower Floor (LF) in room GF3
• Tsif_Z1-Z2 ( °C): Surface Temperature on the upper face of the Intermediate Floor (Z1-Z2-IF)
• Flux_int_Z1-E (W/m2): Flux on Inside face of East wall in Zone 1 (Z1-E)
• Flux_ext_Z1-E (W/m2): Flux on Outside face of East wall in Zone 1 (Z1-E)
• pH_office1_Z1 (W): Power consumption of the heater in Office 1 of Zone 1
• pH_office2_Z1 (W): Power consumption of the heater in Office 2 of Zone 1
• pH_GF1 (W): Power consumption of the heater in room GF1
• pH_GF2 (W): Power consumption of the heater in room GF2
• pH_GF3 (W): Power consumption of the heater in room GF3

Raw data associated with the heatwave climatic scenario, named “Scenario 3″, are given in txt and Excel formats in the supplementary files “Data_scenario3.txt” and “Data_scenario3.xlsx”. The files contain 24 rows:

• Date: Date in Day/Month/Year Hour:Minute
• Tout ( °C): Outside temperature recorded by the weather station mounted on the top of the building
• T_office1-Z1 ( °C): Temperature in Office 1 of Zone 1
• T_office2-Z1 ( °C): Temperature in Office 2 of Zone 1
• Tsi-Z1-S ( °C): Surface Temperature on Inside face of South wall in Zone 1 (Z1-S)
• Tse-Z1-S ( °C): Surface temperature on Outside face of South wall in Zone 1 (Z1-S)
• Tsi-Z1-E ( °C): Surface Temperature on Inside face of East wall in Zone 1 (Z1-E)
• Tse-Z1-E ( °C): Surface Temperature on Outside face of East wall in Zone 1 (Z1-E)
• TGF1 ( °C): Temperature in room GF1 of Zone 2
• TGF2 ( °C): Temperature in room GF2 of Zone 2
• TGF3 ( °C): Temperature in room GF3 of Zone 2
• TGF4 ( °C): Temperature in room GF4 of Zone 2
• Tslf_GF1 ( °C): Surface Temperature on the face of the Lower Floor (LF) in room GF1
• Tslf_GF2 ( °C): Surface Temperature on the face of the Lower Floor (LF) in room GF2
• Tslf_GF3 ( °C): Surface Temperature on the face of the Lower Floor (LF) in room GF3
• Tsif_Z1-Z2 ( °C): Surface Temperature on the upper face of the Intermediate Floor (Z1-Z2-IF)
• Tg ( °C): Temperature of the ground at 0.5 m depth in the vicinity of the building
• Flux_int_Z1-E (W/m2): Flux on Inside face of East wall in Zone 1 (Z1-E)
• Flux_ext_Z1-E (W/m2): Flux on Outside face of East wall in Zone 1 (Z1-E)

**Fig. 5:**
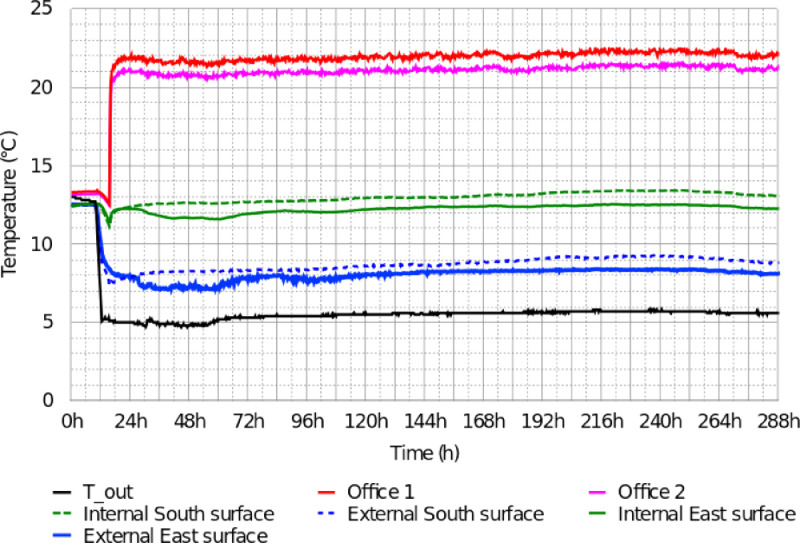
Recorded temperatures in the building – Scenario 1: Stationary conditions, Figure modified from the original research article [1].
• In Fig. 6, the heat fluxes on inside and outside faces of East wall in Zone 1 (Z1-E) from the Scenario 1 (stationary condition scenario);

From the raw data of the different controlled scenarios, we represent:

• In Fig. 5, the temperature sensor outputs from the Scenario 1 (stationary condition scenario). We recall that “Office 1″ and “Office 2″ belong to the open-space on the first floor;

**Fig. 6:**
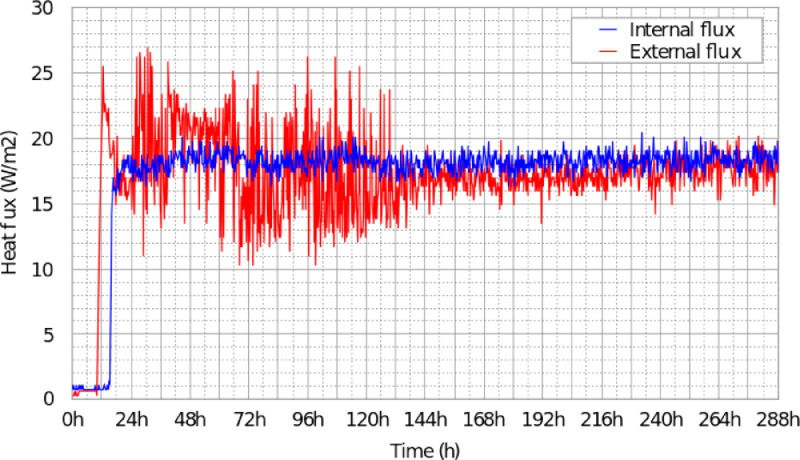
Recorded heat flux on the inside and the outside surfaces of the East-side wall of the building - Scenario 1: stationary conditions, Figure modified from the original research article [1].
• In Fig. 7, the temperature sensor outputs in the building from Scenario 2 (winter climate scenario). As the temperature of the ground was not measured in this scenario, we considered an empirical value of 8 °C;

**Fig. 7:**
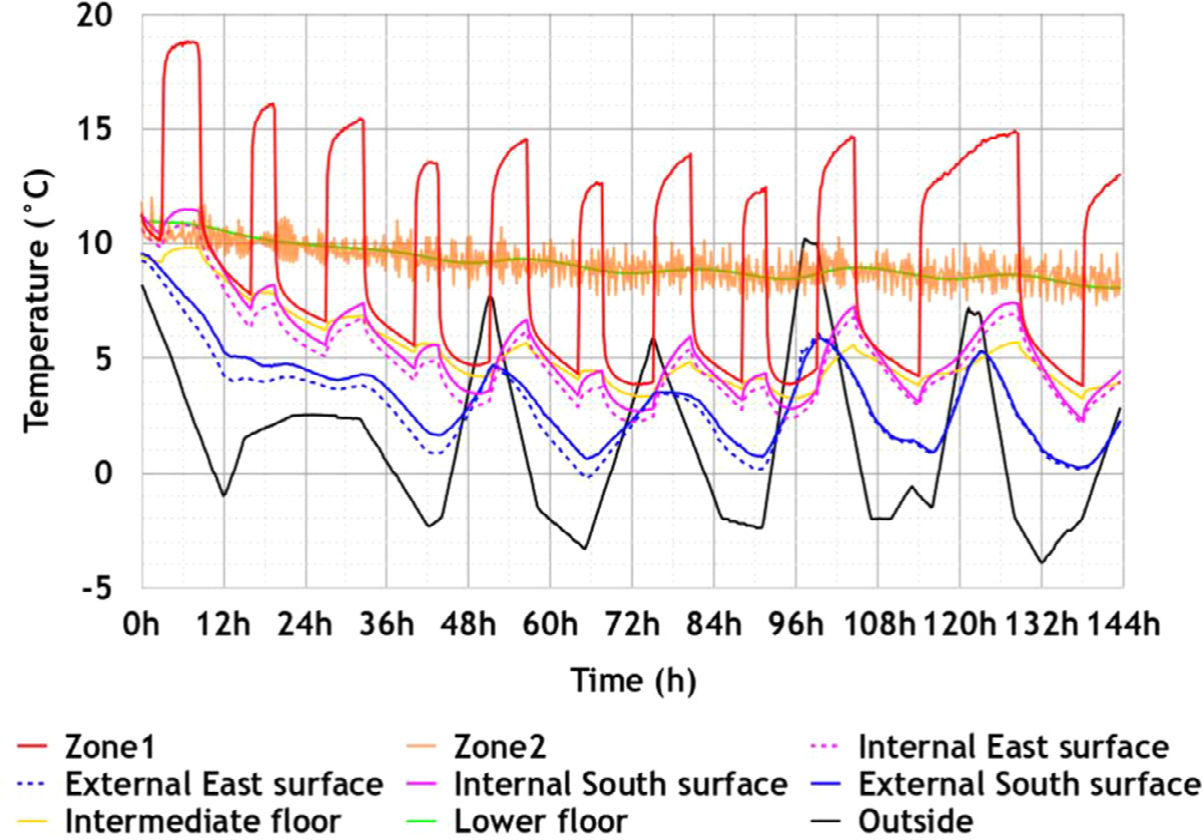
Temperatures measured in the building – Scenario 2: Carpentras winter climate, Figure modified from the original research article [1].
• In Fig. 8, the heat fluxes recorded on the inside and the outside faces of the East oriented wall in Zone 1 (first floor) during the Scenario 2 (winter climate scenario);

**Fig. 8:**
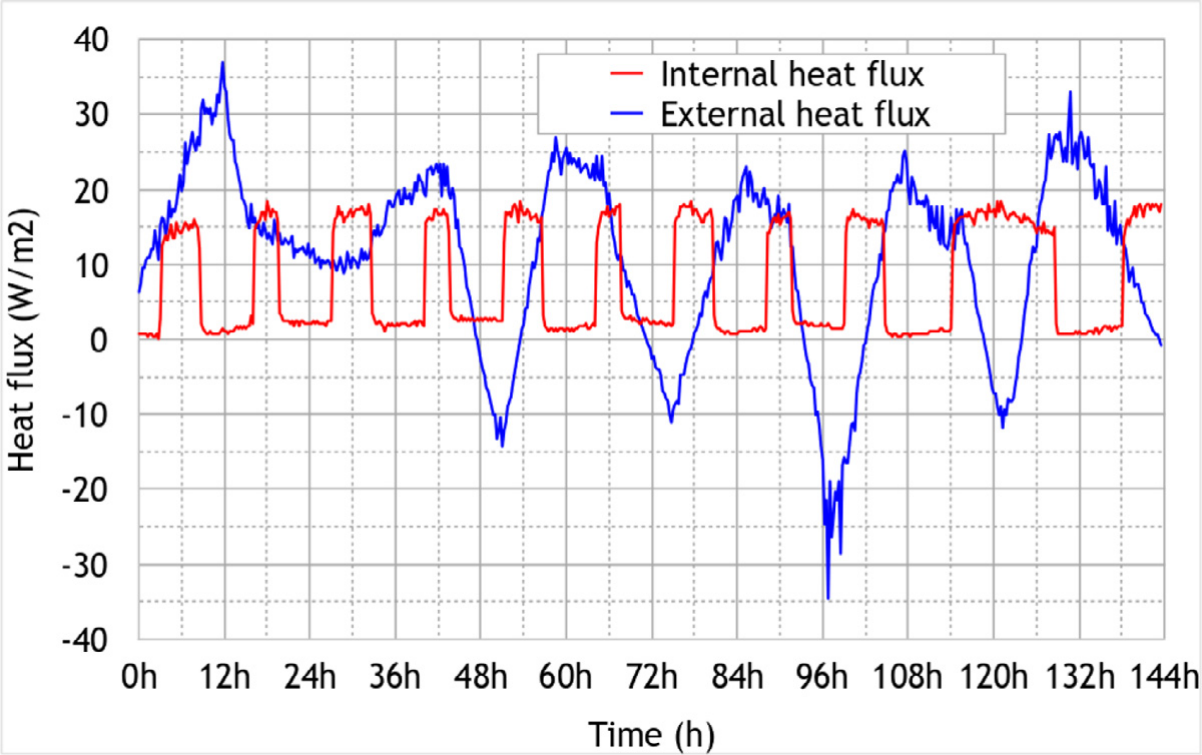
Heat flux measured on the internal and the external surfaces of the East side wall of Zone 1 – Scenario 2: Carpentras winter climate, Figure modified from the original research article [1].
• In Fig. 9, the power consumption of the 5 electric convectors during the Scenario 2 (winter climate scenario);

**Fig. 9:**
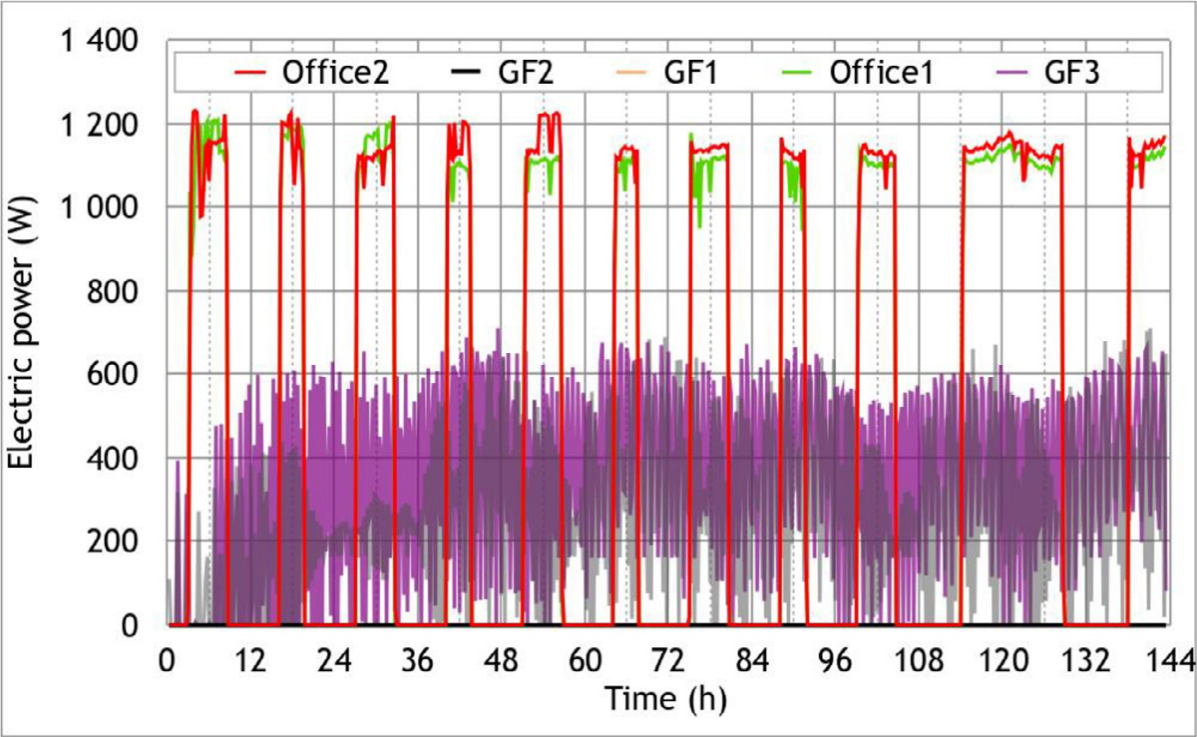
Electrical power used by each heater – Scenario 2: Carpentras winter climate, Figure modified from the original research article [1].
• In Fig. 10, the temperatures measured in the building during the Scenario 3 (heatwave scenario);

**Fig. 10:**
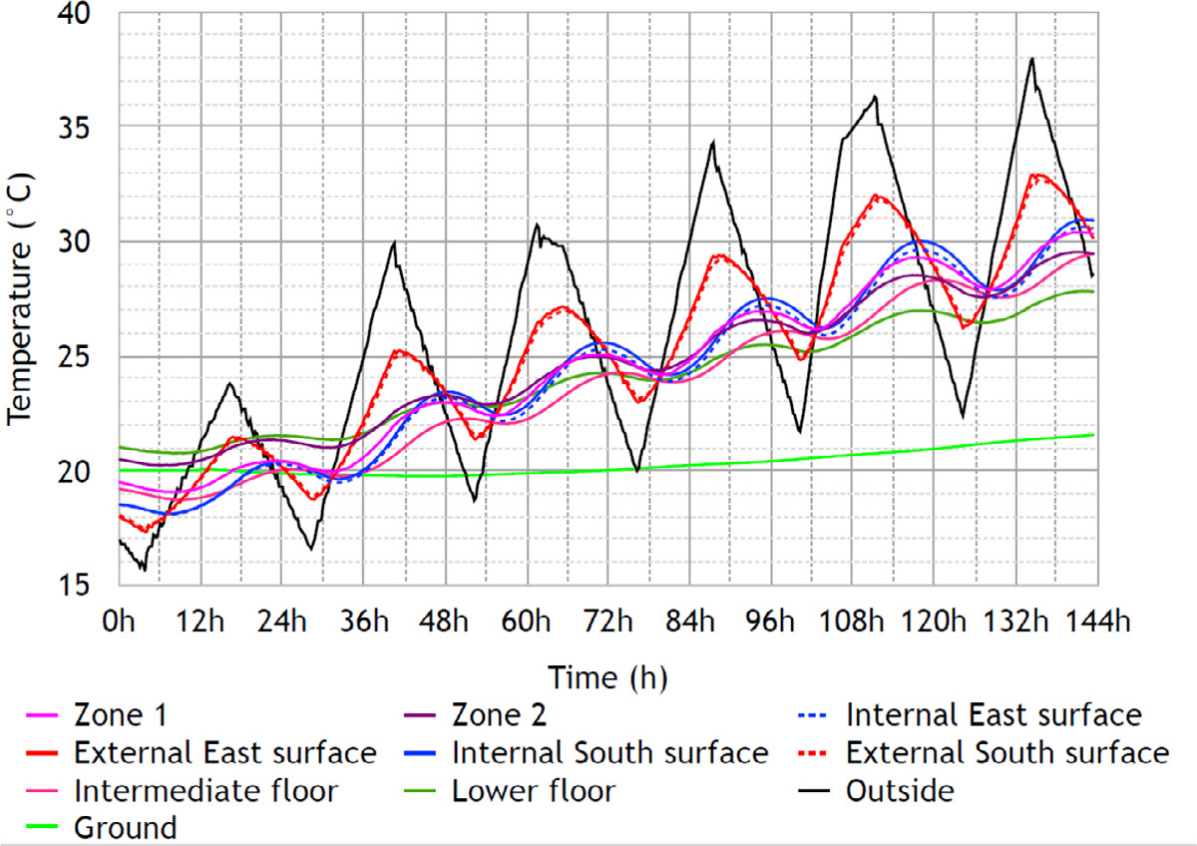
Temperatures measured inside and outside the two-story building – Scenario 3: Paris 2003 heat wave, Figure modified from the original research article [1].

Let us note that the temperature in Zone 1, the temperature in Zone 2 and the surface temperature on the face of the Lower Floor in Zone 2, which are represented in Figs. 7 and 10, are secondary data derived from the raw measured data such that:

• The measured temperature in Zone 1 (first floor) is obtained as the mean value of T_office1_Z1 and T_office2_Z1;
• The measured temperature in Zone 2 (ground floor) is obtained as the weighted average of TGF1, TGF2, TGF3 and TGF4 using the coefficients 0.278, 0.287, 0.305 and 0.130 respectively. These coefficients are determined according to the rooms area;
• The measured surface temperature on the face of the Lower Floor in Zone 2 (ground floor) is obtained as the weighted average of Tslf_GF1, Tslf_GF2 and Tslf_GF3 using the coefficients 0.320, 0.330 and 0.350 respectively. These coefficients are determined according to the rooms area.

## 2 Experimental Design, Materials and Methods

We conducted measurement campaigns to study the thermal behavior of a two-story concrete building that belongs to the “Sense-City” equipment. “Sense-City” is an equipment of excellence founded by the French national research agency (ANR). It consists of a $400\;m^{2}$climatic chamber and two mini-cities of the same size. In practice, the mini-cities are more like small urban districts. The climatic chamber is mounted on rails so that it can be moved from one mini-city to another. It is designed to produce controlled weather conditions by varying the temperature $\left[-10{\;}^{\circ }C;40{\;}^{\circ }C\right]$and the humidity [20%;95%]. It can also reproduce rain and sunshine conditions. The studied two-story concrete building, represented in Fig. 1, belongs to the first mini-city. It has a $40\;m^{2}$area (10m×4m) and 6mheight. Its envelope (walls and floors) is made of 20 cm precast concrete walls. The ground floor consists of 5 rooms (see Fig. 2, Fig. 4 and supplementary file “Building_plan.pdf”). They are all of different areas and usages (see Table 1) and are separated by 72 mm thick concrete walls. On the ground floor, the so-called “GF1” and “GF3” rooms are equipped with a geothermal heating floor that can be fueled by a heat pump. Hence, the composition of the floor is specific in “GF1” and “GF3” for the geothermal system. The water loop in room “GF2” and the heat pump in room “GF3” were switched off during all the experiments. The first floor is an open-space divided into two office rooms (named “Office 1″ and “Office 2″). The wall between the two offices in the first floor was removed in all our experiments. The building is equipped with two controlled mechanical ventilation (CMV) systems (simple and double flow) and an air conditioning system. For our application, only the simple-flow CMV is used. From building technical specifications and 1D anemometer measurement, the forced ventilation was estimated to $112\;m^{3}/h$: $62\;m^{3}/h$on the ground floor and $50\;m^{3}/h$on the first floor. No natural ventilation was considered in all the tests, doors and windows were kept closed.

Three measurement campaigns were conducted in 2019 in the Sense-City equipment to collect the data presented in this article. For each campaign, a different controlled weather scenario was reproduced using the Sense-City climatic chamber. We can underline that controlled climate scenarios were previously examined in another climatic chamber to undertake thermal studies on a typical UK house in [2] and on dwellings in [3]. Let us now describe the three scenarios in the next paragraphs.

Scenario 1: Stationary conditions

In this experiment, the outside temperature was constantly maintained at $5{\;}^{\circ }C$and the humidity at 70%during 12 days of measurement. The geothermal heating floors, the CMV systems, the air conditioning units and the lights simulating the sun were turned off. The building was heated using electric convectors over 12 days to reach stationary conditions. In the first floor (Zone 1), the two convectors with a total power consumption of 2400 W (1200 W each) stayed on for the entire duration of the scenario. The measured temperatures and heat fluxes are respectively presented in Figs. 5 and 6. The sensor outputs are given in txt and Excel format in the supplementary files “Data_scenario1.txt” and “Data_scenario1.xlsx”.

Scenario 2: Carpentras winter climate

This scenario consists of typical winter conditions of a French mild climate region (referenced as “h2d” in the French thermal regulation) represented by the city of Carpentras. Hence, the temperature profile comes from the weather data of the French thermal regulation RT2012. To ensure that the initial state is the same throughout the building, a 12hplateau was added before the experiment began. As with the Scenario 1, the lights simulating the sun were turned off for better control of the test conditions. The simple-flow CMV was in operation for the entire duration of the experiment. Doors and windows were kept closed. On the first floor (Zone 1), we reproduced an occupancy scenario by activating two heaters in the morning from 6am to 9:30am and in the evening from 5pm to 10:30pm (resp. from 8am to 10.30pm) during the weekdays (resp. the weekend). In each room of the ground floor except in the technical room “GF4”, a heater was installed and put in frost protection mode. All the electric convectors were plugged in a connected electrical outlet that allows its remote control and monitoring using Basic R2 sunoff device. The recorded temperatures, heat fluxes and electric consumptions are respectively represented in Figs. 7, 8, and 9 and the sensor outputs are given in txt and Excel formats in the supplementary files “Data_scenario2.txt” and “Data_scenario2.xlsx”.

Scenario 3: Paris 2003 heat wave

The objective of this last scenario was to collect data to study the thermal behavior of the building under summer conditions. Using the climatic chamber of Sense-City, it was possible to reproduce the conditions of the summer 2003 Paris heat wave. As stated previously, the lights simulating the sun were turned off. The simple-flow CMV was in operation for the entire duration of the experiment. Doors and windows were kept closed. Contrary to Scenario 2, in this scenario the ground temperature was measured with a PT100 sensor buried at a depth of 1.5 m in the vicinity of the building. The collected data are represented in Fig. 10 and are available in txt and Excel formats in the supplementary files “Data_scenario3.txt” and “Data_scenario3.xlsx”.

## Declaration of Competing Interest

The authors declare that they have no known competing financial interests or personal relationships which have, or could be perceived to have, influenced the work reported in this article.

## Appendix Supplementary materials

Image, application 1
