# Supplementary material for: Thermal behavior of a two-story concrete building under controlled winter and heat wave scenarios in the sense-city equipment through temperature, flux and energy consumption dataset
Source: Data Brief. 2020 Oct 23;33:106458. doi: 10.1016/j.dib.2020.106458 (PMC7599433; doi:10.1016/j.dib.2020.106458)
Supplement: Supplementary file 1 [file mmc1.zip › Building_plan.pdf]

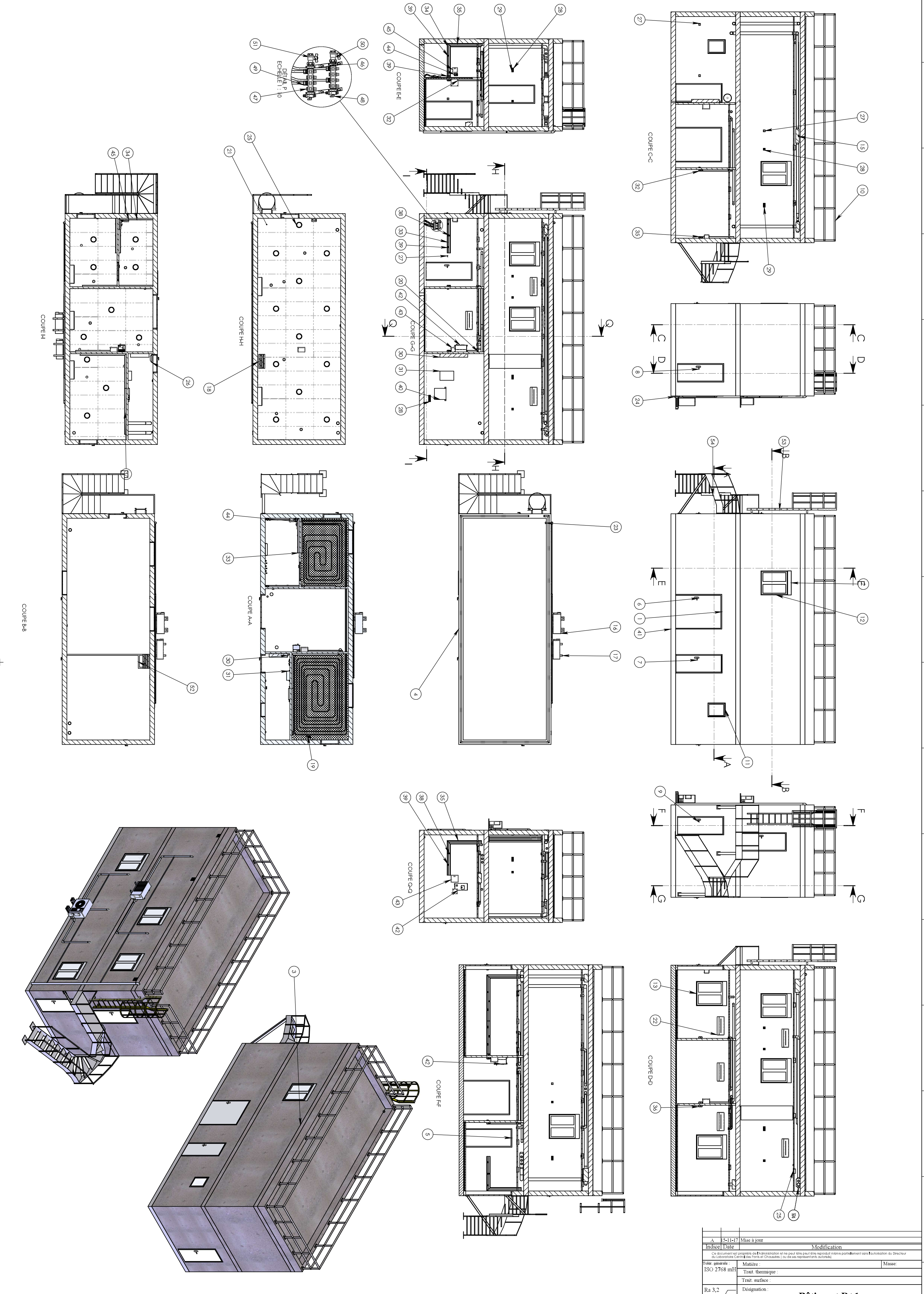

Indice	Date	Modification
A	15-11-17	Mise à jour

Ce document est propriété de l'Administration et ne peut être peut être reproduit même partiellement sans l'autorisation du Directeur du Laboratoire Central des Ponts et Chaussées (ou de ses représentants autorisés)

Tolér. générale : ISO 2768 mH	Matière :	Masse:
	Trait. thermique :	
	Trait. surface :	
Ra 3,2	Désignation : **Bâtiment R+1**	
Echelle : 1:50	IFSTTAR Date : 26/06/2018	Planche 1 : 4 — Dessiné par : E. Merliot — A0 — PLAN N° **908.12.00-1/4**

REP	Mise en plan/Quantité	DESIGNATION	REFERENCE/PLAN	MATIERE	FOURNISSEUR
1	1	90812.003.002_rez-de-chaussée			
2	1	90812.003.003_etage			
3	1	90812.003.004_dalle_toiture			
4	1	90812.003.005_toiture			
5	1	90812.003.010_porte-interieure2(ferme)	200x80cm	POST-FORME	
6	1	90812.003.011_porte_grand_exterieure(Défaut)	200x150 double battant	PVC ou ACIER	
7	2	90812.003.012_porte-exterieure(Défaut)	200x80	PVC ou ACIER	
8	1	90812.003.012_porte-exterieure(Défaut)-2	200x80	PVC ou ACIER	
9	1	90812.003.012_porte-exterieure(Défaut)-3	200x80	PVC ou ACIER	
10	1	90812.003.017_Garde-corps	A0-90812-003-017	Selon choix du type (acier, alu, composite etc...)	
11	1	90812.003.021_petit_fenêtre	75x60cm double vitrage	PVC	
12	4	90812.003.022_grand_fenêtre	Fenêtre à volet roulant intégré 115X100 double vitrage		
13	1	90812.003.022_grand_fenêtre	Fenêtre volet roulant intégre 115X100 triple vitrage		
14	1	90812.003.023_ventilation_simple_flux_etage	A0-908.12.003.023		
15	1	90812.003.024_ventilation_double_flux	A0-90812.003.024		
16	1	90812.003.084_climatisation_RDC	A0-90812.003.084		
17	1	90812.003.085_climatisation_etage	A0-90812.003.085		
18	1	90812.003.080_cloison_movible	ALGAFLEX avec porte		ALGAFLEX
19	1	90812.003.105_system_plancher_chauffant	A0-90812.003.105		
20	1	90812.003.082_faux_plafond_RDC	PERLA OP 0,95 1200x600x15mm + Rail BOARD		AMSTRONG
21	1	90812.003.083_faux_plafond_etage	PERLA OP 0,95 1200x600x15mm + Rail BOARD		AMSTRONG
22	5	90812.003.088_monosplit	MSZ-SF25VE2		MITSUBISHI ELECTRIC
23	1	90812.003.103_coude pluvial	Coude PVC DN80	PVC	
24	1	90812.003.104_tuyau_eau_pluvial	Tube PVC DN80	PVC	
25	22	90812.003.113_lampe	DN5708-LED12S		PHILIPS
26	1	90812.003.113_lampe_mural	Coreline Hublot WL 120V LED12S		PHILIPS
27	7	90812.003.114_interrupteur	PLEXO 10AX 250V		LEGRAND
28	13	90812.003.115_prise_electrique	PRISE 2P+T PLEXO		LEGRAND
29	4	90812.003.116_prise_ethernet	RJ45 PLEXO		LEGRAND
30	1	90812.003.117_tableau_electrique_distribution_principale	A concevoir par le titulaire selon CCTP		
31	1	90812.003.118_tableau_electrique_220V	A concevoir par le titulaire selon CCTP		
32	4	90812.003.121 Goulotte	Goulotte 2 compartiments 130x50 MOSAIC		LEGRAND
33	2	90812.003.121 Goulotte	Goulotte 2 compartiments 130x50 MOSAIC		LEGRAND
34	1	90812.003.121 Goulotte	Goulotte 2 compartiments 130x50 MOSAIC		LEGRAND
35	6	90812.003.121 Goulotte	Goulotte 2 compartiments 130x50 MOSAIC		LEGRAND
36	1	90812.003.121 Goulotte	Goulotte 2 compartiments 130x50 MOSAIC		LEGRAND
37	1	90812.003.121 Goulotte	Goulotte 2 compartiments 130x50 MOSAIC		LEGRAND
38	8	90812.003.122 Prise Ethernet	Prise RJ45 MOSAIC goulotte		LEGRAND
39	22	90812.003.123 Prise 220V 2P+T	Prise électrique 2P+T 250V MOSAIC goulotte		LEGRAND
40	1	90812.003.125 Switch fibre optique RJ45			
41	1	9080706 Dalle immeuble	A0-908.07.06	Béton Armé selon calcul structurel	
42	1	90809124 Variateur de vitesse ALTIVAR-61-15kW	ATV61E5D15N4		Schneider Electric
43	1	90809125 Coffret regulation niveau cuve	NSYS3DEX3320		Schneider Electric
44	1	90818108 Variateur de vitesse ALTIVAR_312_220V_1,5kW	ATV312HU15M2		Schneider Electric
45	1	90818111 Coffret commande variateur de vitesse 1,5kW	A concevoir par le titulaire selon CCTP		
46	2	90812100 Support fixation collecteurs double	658200		CALEFI
47	2	90812101 Collecteur a vanne 4 voies 354	6630		Caleffi
48	2	90812102 Extremité collecteur 599675	599675		CALEFI
49	4	90812104 Raccord union droit 9402304 PER D16	9402304		COMAP
50	1	90812103 Vanne d'arret 391077	391077		CALEFI
51	1	90812103 Vanne d'arret 391077	391077		CALEFI
52	1	90812.003.006_dalle20cm			
53	1	90812.003.128 Echelle à crinoline H2800_			
54	1	90812.003.008_escalier_exterieur_V2			

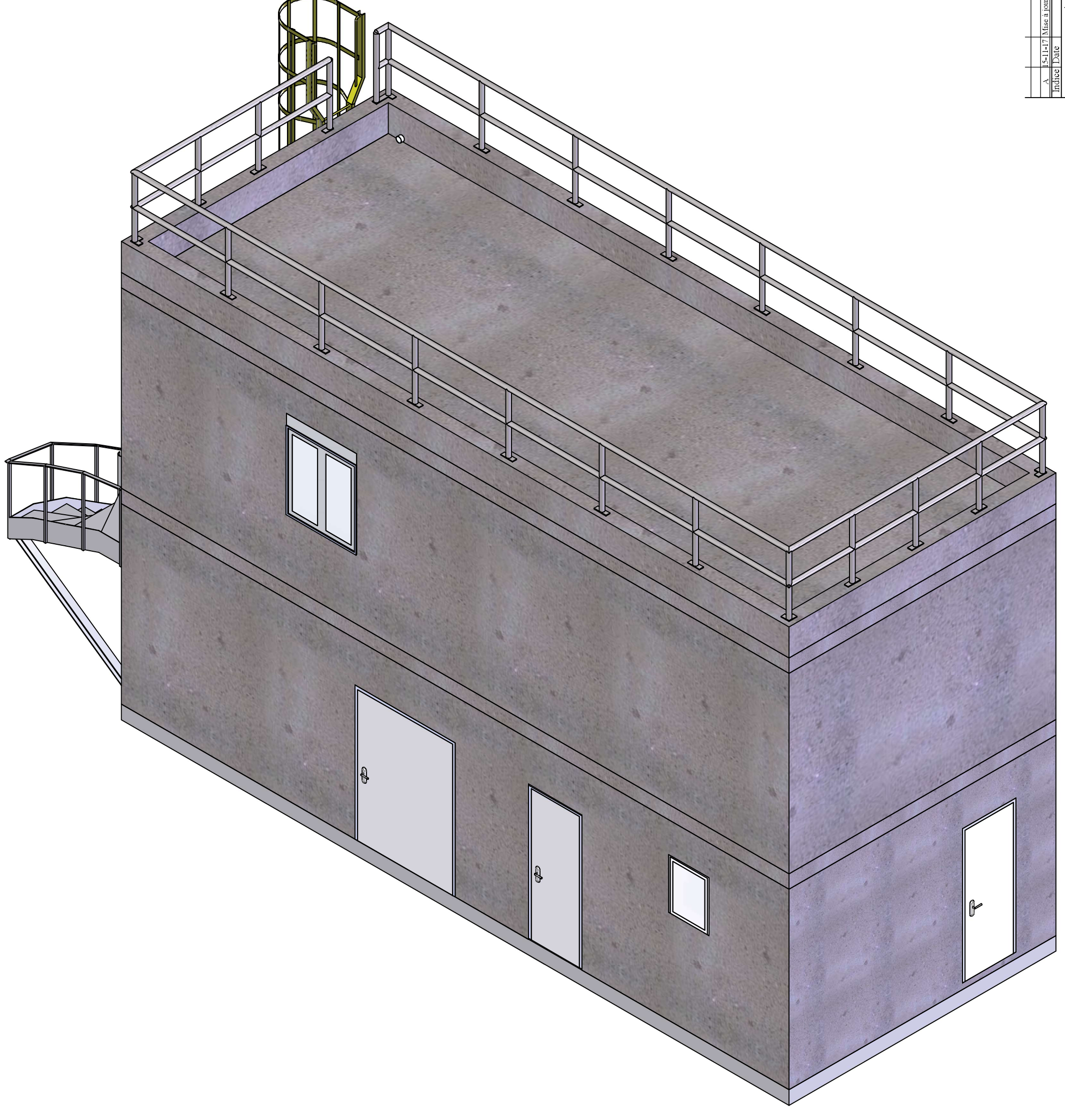

Bâtiment R+1

Planche 2 : 4 — A0 — PLAN N° 908.12.00-2/4

Echelle 1:20 — IFSTTAR — Date: 26/06/2018

Ra 3,2 — Tolér. générale: ISO 2768 mH

Les surcharges sont indiquées pour le calcul structurel du bâtiment et du radier

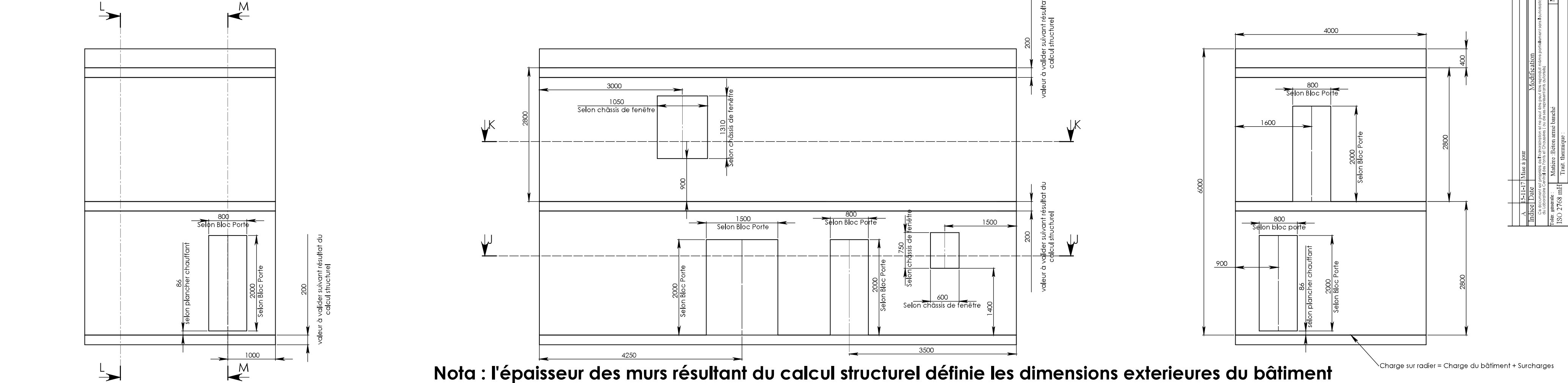

Nota : l'épaisseur des murs résultant du calcul structurel définie les dimensions exterieures du bâtiment
Si l'épaisseur calculée est supérieur à 200mm les cotes du radier et les cotes extérieures du bâtiment seront ajusté en concéquence pour concerver les dimensions intérieures des pièces.
Si l'épaisseur est inférieure à 200mm les cotes du radier et les cotes extérieures du bâtiment seront celles indiquées sur le plan et les cotes intérieures des pièces seront ajustées.

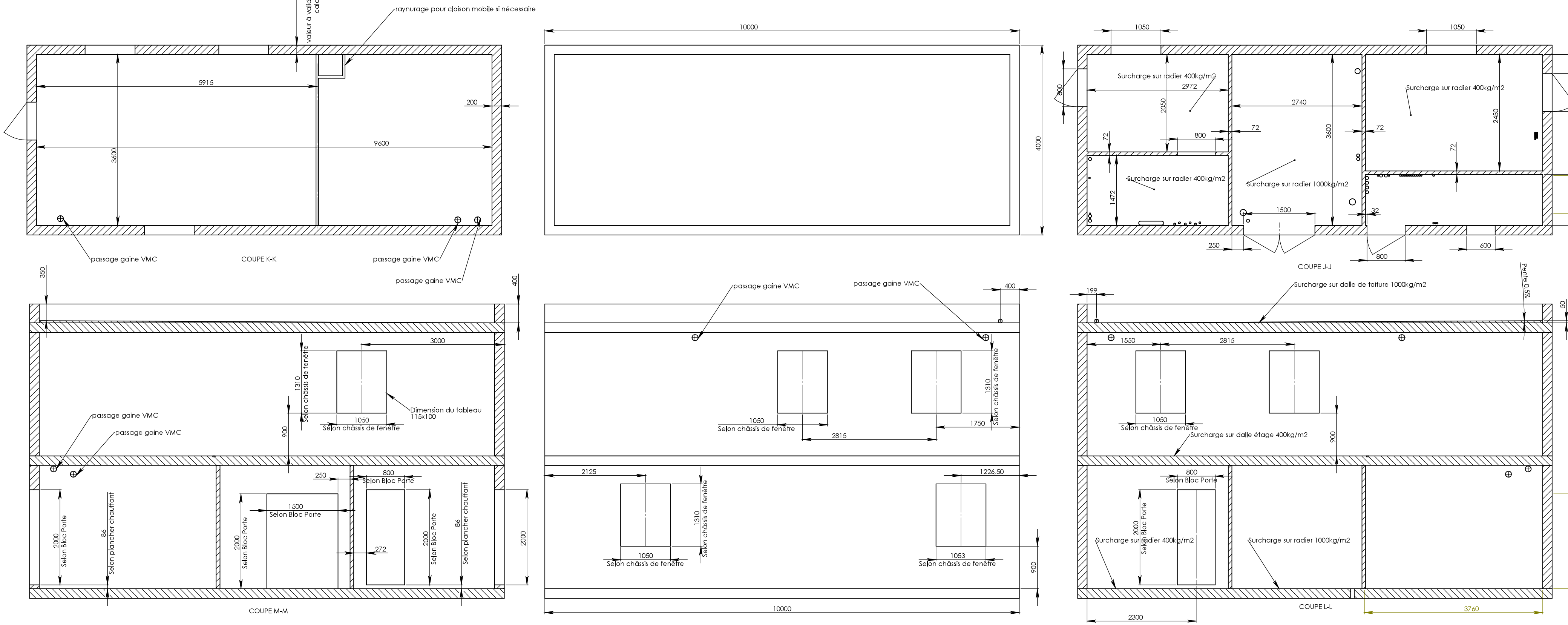

Structure du bâtiment
Planche 3 : 4 — A0
PLAN N° 908.12.00-3/4
IFSTTAR
Échelle 1:30
Matière : Béton armé banché
Tolér. générale ISO 2768 mH
Dessiné par E. Merlhot — Date : 26/06/2018
A — 15-11-17 — Mise à jour
Indice — Date — Modification

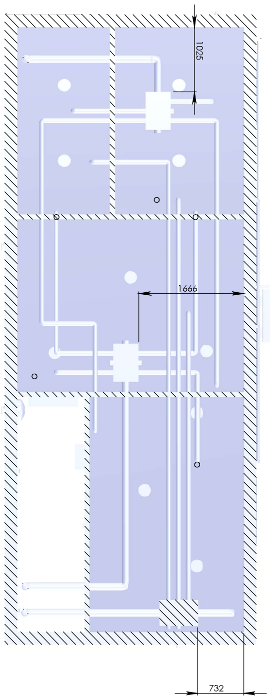

COUPE O-O

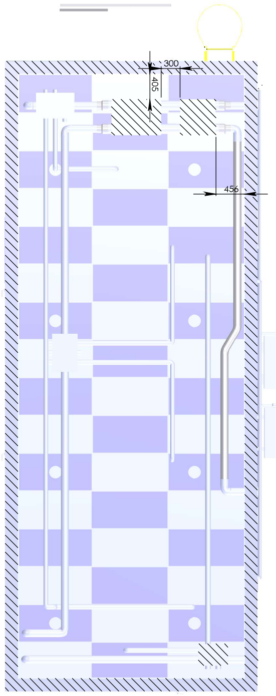

COUPE N-N

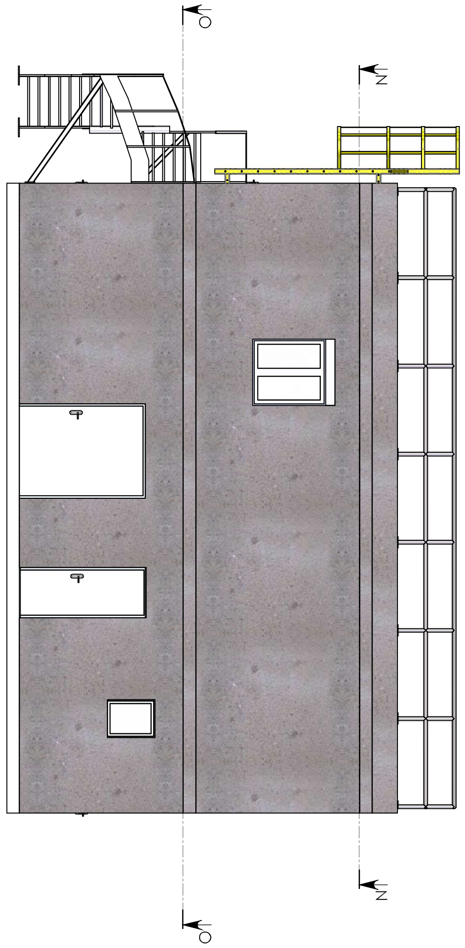

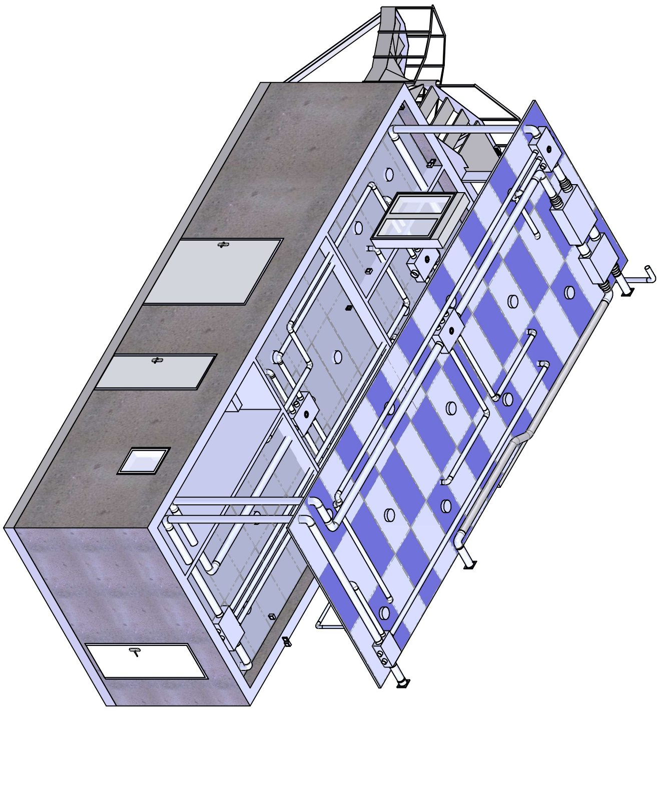

A	15-11-17	Mise à jour
Indice	Date	Modification

Ce document est propriété de l'Administration et ne peut être peut être reproduit même partiellement sans l'autorisation du Directeur du Laboratoire Central des Ponts et Chaussées (ou de ses représentants autorisés)

Tolér. générale : ISO 2768 mH	Matière :	Masse:
	Trait. thermique :	
	Trait. surface :	
Ra 3,2	Désignation : **Bâtiment R+1**	
Echelle : **1:30**	IFSTTAR Date : 26/06/2018	**Planche 4 : 4** — Dessiné par : **E. Merliot** — A0 — PLAN N° **908.12.00-4/4**
